# Parents’ Experiences of Suicide-Bereavement: A Qualitative Study at 6 and 12 Months after Loss

**DOI:** 10.3390/ijerph15040618

**Published:** 2018-03-28

**Authors:** Victoria Ross, Kairi Kõlves, Lisa Kunde, Diego De Leo

**Affiliations:** Australian Institute for Suicide Research and Prevention, WHO Collaborating Centre for Research and Training in Suicide Prevention, Menzies Health Institute of Queensland, Griffith University, Mt Gravatt Campus, Mount Gravatt, QLD 4122, Australia; victoria.ross@griffith.edu.au (V.R.); l.kunde@griffith.edu.au (L.K.); d.deleo@griffith.edu.au (D.D.L.)

**Keywords:** suicide-bereavement, parents, sense-making, meaning-making, coping, qualitative

## Abstract

The death of a child by suicide is a severe trauma, placing parents at greater risk of psychological morbidity and physical health problems compared to other causes of death. However, few studies have examined the aftermath and bereavement experience for parents following the death of a child to suicide, limiting the ability to guide effective postvention services through empirical research. The current study, which was part of a larger longitudinal investigation of suicide bereavement in Queensland, Australia, examined the individual experiences of both mothers and fathers bereaved by suicide over time, specifically at the six month and 12 month time points after their loss. Bereaved parents who had provided written consent to be contacted for research purposes were identified through the Queensland Suicide Register, and took part in individual, semi-structured interviews. Generic qualitative analysis identified three key themes: searching for answers and sense-making, coping strategies and support, and finding meaning and purpose. Some participants showed indications of meaning-making and post-traumatic growth at 12 months after the suicide. According to the dual process model of bereavement, it is likely that participants were still oscillating between sense-making and meaning making, indicating that adapting to bereavement is a dynamic and fluctuating process.

## 1. Introduction

Suicide is a tragic event that has a devastating and far-reaching impact on those left behind. By current World Health Organization data, approximately 800,000 people die by suicide annually [1]. In a recent meta-analysis estimating the prevalence of those exposed to suicide, Andriessen, Rahman, Draper et al. [2] calculated that 4.3% and 22% of the population have been exposed to a suicide during the past year and their lifetime, respectively. In addition, in a study by Berman investigating the population of people impacted by suicide, parents of children who had died by suicide estimated that more than 80 individuals would have been intimately and directly affected by their own child’s suicide [3].

The death of a child by suicide is a severe trauma, placing parents at greater risk of psychological morbidity and physical health problems compared to other causes of death [4,5]. Moreover, mothers, but not fathers, who have lost a child to suicide are at a greater risk of suicide than mothers bereaved by other causes [6]. These findings are mainly from large-scale studies that do not give a clear picture of the individual pathways of parental bereavement, highlighting the need to complement this research with qualitative analyses of parental journeys.

Previous qualitative research has studied the impact of suicide bereavement on adults, including family members and close friends who had a range of relationships to the deceased [7,8,9]. However to date, few studies have qualitatively examined the aftermath and bereavement experience for parents following the death of a child to suicide [10,11], and consequently the ability to guide effective postvention services through empirical research is limited. The current study aims to examine the individual experiences of both mothers and fathers bereaved by suicide over time, specifically at the six month and 12 month time points after the death of their child.

## 2. Materials and Methods

This study is part of a larger longitudinal investigation of suicide bereavement over two years in Queensland, Australia [12]. The investigation included both quantitative and qualitative components. In the frames of the current document, we present the qualitative results based on interviews with parents bereaved by suicide at six and 12 months after their loss. These time-points were selected for practical purposes as ethically; six months post-bereavement is the earliest time in which suicide-bereaved people may be contacted.

### 2.1. Data Collection

People bereaved by suicide were identified utilizing the Queensland Suicide Register (QSR), a suicide mortality database managed by the Australian Institute for Suicide Research and Prevention (AISRAP). Bereaved parents who had provided written consent to be contacted for research purposes were sent a letter from an AISRAP clinical interviewer approximately five months after the fatal event. Approximately two weeks after sending the letter, the clinical interviewer contacted participant by telephone, inviting them to participate and to arrange a time and place for the interview. The participants were sent another letter at 11 months to schedule an interview for approximately 12 months after their loss. Interviews in the frames of the pilot study were conducted in 2012 [12] and in the frames of the large-scale study in 2014–2016.

Semi-structured interviews were conducted either by telephone in a confidential room or face-to-face in an environment comfortable to the participant. Interviewers adopted a facilitative and neutral approach where participants were encouraged to talk at length in their own words without interruption and with minimal prompting. Initial interviews lasted approximately two and a half hours and follow-up interviews, two hours. A short introduction and then debriefing were conducted at the commencement and at completion of the interview, allowing participants’ concerns, queries or feelings to be addressed sensitively. The initial, six month interview followed a topic guide consisting of the following sections: (1) a qualitative component—consisting of a few open-ended questions about the events leading to the death and parents’ feelings and experiences since that time; and (2) a quantitative component—which collected sociodemographic information; medical and psychiatric history, including suicidal behavior of deceased and bereaved; and post-event experiences measured with different validated scales [12]. The follow-up, 12 month interview focused on questions about life events and changes since the initial interview. Interviews were conducted by trained female clinical interviewers, with postgraduate health qualifications (psychology, social work or nursing). All interviewers were skilled and experienced in working with the bereaved, and proficient in recognizing participants’ needs and providing assistance should aparticipant become distressed. All interviews were audiotaped with the participant’s consent, then transcribed verbatim. Pseudonyms were allocated for childrens’ names in order to ensure confidentiality.

### 2.2. Participants

Purposive sampling was conducted from the responses of the 49 mothers and 24 fathers who participated in the large-scale study. Factors considered in sampling included representation of both mothers and fathers, gender of the child, age of parent and child and help seeking (yes/no). Parents also had to have participated in both interviews to be included in the sample. Cases were included until data saturation was reached. The final sample was comprised of seven mothers (M age = 60.1 years, range: 50–78) and seven fathers (M age = 59.9 years, range: 50–68). Ten parents were bereaved of their sons and four were bereaved of daughters (M age = 29.3 years; range: 15–51). One parent of each deceased child was included in the sample (i.e., there were no couples).

All participants were provided with written information, which included the identity and affiliation of the facilitators and researchers, aims of the research, and confidentiality and informed consent issues, including the right to withdraw voluntarily. All participants were required to provide written consent. The study was approved by the Griffith University’s Human Research Ethics Committee (CSR/04/11/HREC).

### 2.3. Data Analysis

This study employed a generic (inductive) qualitative approach with a focus on human experiences [13]. Braun and Clark’s [14] five step process for conducting thematic analysis was followed. NVIVO 11 (QSR International, Melbourne, Australia) was utilized to manage the data. Initial coding was carried out by V.R. and L.K. (both psychologists). Steps were taken to ensure validity of the analysis—coding procedures were continuously discussed with K.K. (a sociologist), which allowed the reassessment of themes and interpretations until consensus was reached. Themes were chosen on the basis of universality and salience rather than on prevalence alone [14]. The study was reported according to the Consolidated Criteria for Reporting Qualitative Research (COREQ) checklist criteria for reporting qualitative research [15].

## 3. Results

Three key themes were identified from the analysis: searching for answers and sense-making, coping strategies and support, and finding meaning and purpose.

### 3.1. Searching for Answers and Sense-Making

All participants (male and female) described their struggles to make sense of their loss, and their search for answers for reasons for their child’s suicide. This contemplative and reflective process was dominant across all interviews at both six months and 12 months. Parents spoke of their often traumatic experiences leading up the suicide, such as their child’s mental health problems, incidents of self-harm and suicide attempts; and in turn, questioned what could have been done differently. Where there had been no previous indications that the suicide would occur, parents described their feelings of shock and bewilderment, and reflected on their many unanswered questions about the motivations for the suicide. Some parents described their frustrations at trying to obtain information from coroners, psychologists and doctors in order to gain some understanding of the reasons for the suicide. For some, the process of searching for answers resulted in anger and blaming others (e.g., their child’s friends who were perceived to be a bad influence, doctors and the health system that were not able to help).

“You question so much all the time. Because you’re going to naturally question whether it’s you, whether he’s in trouble at uni, money trouble… Maybe he was depressed. I don’t know. We didn’t see any signs... It would’ve been nice to have someone who would’ve had the answers, to tell you the thought processes that could go on. But no one’s really had any idea. Just the questions behind why—give us some ideas why he would’ve done it.”

(Father: 6 months)

“There are times when you start to think and you think, why? I mean we had no idea that he’d ever do anything like this, we didn’t think he would. He even said that he would never ever do anything like this, and then to turn around and do it.”

(Mother: 6 months)

I don’t think they did the right thing for her. They were treating her and checking her out of hospital five days later into the same environment where she came from. Is this the right way to treat these sorts of people? I suppose I’ll always question why the medical system had to let her down. I’m looking for somebody to blame, somebody’s ass to kick. How did this happen? What could you do to prevent it?

(Father: 12 months)

Despite their search for answers and struggles to make sense of their loss, several mothers at both the six and 12 month period, indicated that they were beginning to accept the finality of the death of their child and were resigned to the fact that the situation could not be changed.

“My answers probably would have been different six months ago, but now I’m, like I’m resigned to the fact that she’s not coming back obviously. It’s just, as time passes the pain doesn’t go away but it gets easier.”

(Mother: 6 months)

“We’ve gone through a year and you still have moments of why and if, and all the rest of it, but I suppose in my head and in my heart I know that it doesn’t matter what I do now, she can’t ever re-arrive. It’s not like in the beginning when you think ‘oh it’s like as if she’s gone away and she’s still alive’, but you realize after a while that doesn’t happen… but it leaves a massive void. There’s a deep spot there and we know that place in your head and heart will become less painful, but that spot still just remains.”

(Mother: 12 months)

### 3.2. Coping Strategies and Support

Parents revealed a variety of coping styles—both adaptive and maladaptive, although there were no obvious differences between mothers and fathers in the types of strategies applied. A number of parents mentioned avoidance of the topic (i.e., refusing to discuss the loss of their child with their partner/families or others), with some individuals describing how they did not like to talk about the suicide to their partner, and others recounting how it was their partner who refused to discuss their loss. These examples were seen across both genders. Another example of avoidance was shown in fathers who reported working excessively in order to avoid the pain of thinking about their loss. Several parents also mentioned that they were drinking excessively, and for some this problem appeared to be increasing. Others spoke of their difficulties sleeping and their subsequent use of alcohol and/or marijuana in order to help them sleep at night. One mother described how she was only just coping, and described her pain as something that she simply had to endure.

“But we don’t really talk about it—if you mean the incident or what happened.”

(Father: 12 months)

“It’s the weekly, every day drinking in the week that’s definitely increased. Whereas before, we’d try not drink for three days … but now it’s definitely, at least one bottle to myself, every night.”

(Father: 6 months)

“Like I said, you know, you either collapse under the pile, or you scrabble up with it, dig in your toes, and your fingernails, and even your teeth if you have to, to just rise above it …”

(Mother: 6 months)

There were also numerous examples of adaptive coping strategies implemented by bereaved parents. Parents described positive coping strategies that ranged from simple approaches such as trying to maintain a positive attitude and looking after their physical and mental health, to more complex strategies such as keeping memories alive and rituals that helped ensure a continuing bond with their child. Several fathers told of how they found it helpful to keep a journal where they wrote letters to their child. Some parents described the importance of celebrating their child’s birthdays (which was generally thought to be far more positive and preferable to marking the day of their loss). Others maintained a connection with their child through visits to their loved one’s gravesite or resting place. Some parents (both male and female) described how their faith/religion and attending church had helped them cope. Keeping occupied and maintaining a routine through work and other interests were also cited as coping strategies.

“If I was to say there were two things that have helped a lot in me just processing what’s going on in life and where I’m at and reflect on myself—going for walks and thinking and a little bit of talking out loud … But also, writing a kind of journal, which is typically just like a brain dump of where I’m at, what I’m thinking. On occasions, I’ve done a letter to Edward and they’ve been good ways for me to step back and evaluate where I’m at.”

(Father: 12 months)

“I go (to the cemetery) every week. I pick up the flowers on a Friday because I’ve got a standing order at the florist.”

(Mother: 12 months)

“My sister made this amazing cake which somehow she managed to put a vibrant pink heart through the centre. So again, the family came together in little dribs and drabs to celebrate.”

(Mother: 6 months)

Some parents (both mothers and fathers) described how attending individual counselling and suicide bereavement support groups had been critical to their ongoing coping and recovery. When discussing support groups, participants spoke of their sense of relief at being able to talk to others who understood and had suffered the same loss. In addition, one mother recounted how her workplace had been greatly supportive of her loss (e.g., helping her to access counselling, and showing empathy and consideration regarding her need to take time off), which she felt helped with her ability to cope.

“We found it very useful … everyone tells their story and you can open up and they tell you things. You stop feeling like you are the only unlucky people in the world. That it does happen to other people as well, even if it’s a small number. You’re not the only ones, which is comforting to know that there’s other people (in the same position).”

(Father: 6 months)

“I see him (psychiatrist) about once a month and I just sort of, I suppose, put all the questions into my head that I think need to be answered and of course he doesn’t answer them, I answer them, but it’s something to discuss together.”

(Mother: 12 months)

There was considerable variation in levels of support received by bereaved parents, with some participants describing strong support from their partner/spouse, family and friends, and others with very little support. Several individuals said they felt fortunate to be able to speak openly and share feelings with their partners and family members, and described how this helped with their ability to cope. One father spoke of the importance of friendships and having a sense of connectedness to others. However, a number of others reported feeling less patient and uncomfortable with family and friends, resulting in their withdrawal from social interactions; indicating reduced opportunities to receive social support.

“I’ve got a good supportive family group. I think I have a good enough family and friendships to be able to share thoughts and feelings … and I can talk openly with.”

(Father: 12 months)

“Even if I go to my friends’ houses and I see their family together it upsets me a little bit … and then someone says ‘how many kids have you got?’ and I’ve got to say ‘four now’(referring to the loss of her son).”

(Mother: 12 months)

“When I go to my outer family … if anything I’ve probably shut down a bit more to them. I’ve probably become a bit more insular.”

(Mother: 12 months)

### 3.3. Finding Meaning and Purpose

At the 12 months interviews, a number of parents indicated that they had come to terms with their loss and had started to find meaning and purpose in their lives. For several parents, living through their loss and grief was seen as a learning process which had led to some positive outcomes. Participants spoke of how their experience had made them reflect and re-evaluate their lives, which had in turn enabled them to grow emotionally and spiritually. In addition, many parents described how they felt that they had a greater awareness of others who might be in need of help, and were more aware and open to listening and offering help.

“For me it was cathartic. It helped me have a purpose. As I say I’ve probably readdressed quite a few things in my life, or we both have. Helping people go forward, to me is a great thing and certainly helping any young person deal with the un-assurances of life.”

(Mother: 12 months)

“I just attribute this sense of reinvigorated connections with other people, this much more candid and open and openly caring kind of two-way relationships that I’ve got with all these people, including my close family, have really come about because I got this big wake-up call from Edward leaving. It just catapulted me into a different way of living. It’s like it’s just made me do this whole reprioritising and re-evaluating.”

(Father: 12 months)

Others spoke of how they had learnt to change their priorities, placing more value on life and not taking everyday things for granted. Participants described a wide variety of ways in which they were making their lives meaningful again to enable them to move forward. Making a positive contribution through work, helping others through charity work and fundraising, connecting with nature through walks and camping trips, and simply being open to enjoying experiences and friendships were all cited as ways in which parents were beginning to move forward with life. However, it should be noted that not all parents reported such positive responses, with some still struggling with their grief and unable to move forward at the 12 month time period.

“I’m definitely living life. I feel sorry for other people that are just surviving because I’m going through everything at the moment, but it’s a privilege. I’ve always been a bit spiritual, but I’m making more decisions now and I’ve grown up. I’ve said to (my wife) this is it; it’s not going to be ‘we’re going to be good to each other today and then tomorrow we’re not’. It’s going to be forever and that’s it. It’s just being happy and living your life. And that’s because of Peter, exactly because of Peter.”

(Father: 12 months)

“It doesn’t matter whether you have two or 10 or 20 (years of life left) the lesson that it teaches you is make the best, look into it and see what’s really good in there and take that out and go with it. Make sure that the things that you’re doing right have purpose. Don’t dilly-daddle with the nonsense in life, you know. You know life is very precious and you have to do the best with the time you’ve got, whatever that time is.”

(Mother: 12 months)

“I have good days and bad days. It’s horrible, just horrible. There’s probably not a day goes by that I don’t have a cry ... It just doesn’t get any easier.”

(Mother: 12 months)

“I tend to think more about being deliberate in how I use my time to enjoy it and to take in new experiences, or revisit experiences I’ve enjoyed that I might have let fall by the wayside over the years. So a drive to the beach, a walk on the beach, trip in the country, just being on the open road, all those are things that I haven’t done much in recent years.”

(Father: 12 months)

## 4. Discussion

This study identified three key themes in parental responses to suicide bereavement: searching for answers and sense-making; coping strategies and support; and finding meaning and purpose. The prominence of engaging in rumination and searching for answers is consistent with current literature on the experiences of the suicide-bereaved and their need to make sense of their loss [10,16,17,18]. Researchers suggest there are two types of rumination: a sense-making phase, followed by a meaning-making phase which initiates positive growth [17,19]. According to Moore et al. [18], the question of ‘why?’ must be satisfied by parents in a way that makes sense and is acceptable to them, and they may need to revert between ‘brooding’ and effective rumination until this occurs. Genest and colleagues [17] suggest that sense-making refers to understanding and accepting that the suicide has occurred, while meaning-making refers to finding a positive response to the suicide that leads to post-traumatic growth. At both the six and 12 month time points in this study, several mothers indicated that they had found acceptance of the loss of their child, suggesting that they had reached the sense-making component of this rumination phase.

Coping strategies and levels of support varied considerably among parents in our study. A range of both maladaptive and adaptive strategies were described—from avoidance (not discussing the death, excessive work and alcohol use) to maintaining physical and mental health, and rituals such as writing letters and celebrating birthdays to ensure continuing bonds with the child. Where the prevailing thought was once that grief should be resolved by disconnecting from the deceased, new models of grief and loss allow for ongoing relationships and emotional connections, for example supporting ongoing rituals and marking of special occasions [20,21]. Research now suggests that continuing bonds with a loved one may have an adaptive function through the maintenance of a psychological rather than physical bond [10,22]. In our study, parents found it important to maintain psychological bonds with their child through rituals.

Reports by some parents of social withdrawal and subsequent isolation and reduced social support are consistent with existing research on suicide bereavement [10,16]. Shields et al. [10] suggests that support groups may help fill the void where parents bereaved by suicide are lacking family or social support. Consistent with the literature, participants in this study reported their feelings of comfort and relief in being understood and talking to others whom had shared experiences [10,23]. The current research supports the notion that support groups may play a crucial role in suicide-bereaved parents’ ability to make sense of their loss and reconstruct their lives in a helpful way [10,24]. An important issue identified through studying individual parents’ bereavement experiences was that a number of parents had difficulties in sharing their feelings and/or talking about the loss of their child with their partner. Suicide-bereavement support groups may also offer opportunities for individuals in this situation to share their feelings in a supportive and understanding environment.

Parents’ descriptions of finding meaning and purpose at 12 months after the suicide of their child indicates the meaning-making process and the positive transformation and learning associated with post-traumatic growth. Parents’ descriptions of an increased sensitivity to peoples’ needs, becoming closer to others, and greater appreciations of friendships and of life in general are also consistent with descriptions of post-traumatic growth in the literature [17,24]. Researchers suggest that that the process of meaning-making is critical to suicide-bereaved parents as it enables them to go on living in a world that no longer contains their child [10,25]. While meaning-making is associated with healing and post-traumatic growth, the inability of the suicide-bereaved to find meaning within the death could impact on healing and may lead to complicated grief symptoms [8,10,22,26]. It should be noted that not all parents in our study described finding meaning or indications of post traumatic growth.

The three themes identified in this study appear to be well explained by the dual process model of coping with bereavement [27]. According to this model, there are two categories of stressors associated with bereavement. Loss-oriented stressors, which involve processing of the loss experience and searching for answers, clearly link to the sense-making phase experienced by our participants. Moreover, restoration-oriented stressors, which reflect rethinking and replanning one’s life in the face of bereavement, corresponds with the meaning-making phase of bereavement. The model suggests that the coping strategies of the bereaved, both adaptive and maladaptive, oscillate between the two processes. According to Stroebe and Schut [27], the principle underlying oscillation is that at times the bereaved will confront aspects of loss and restoration, and at other times avoid them. Oscillation between the two types of stressors is necessary for adaptive coping (e.g., positive reappraisals are important for moving forward, but if these are constantly maintained, then rumination and coming to terms with the loss is neglected) [27].

Interestingly, the indications of post-traumatic growth seen in our participants at 12 months after the suicide are not consistent with the literature which indicates that personal growth tends to drop in first year after a loss, and then rises steadily as the years pass after the loss [24]. The application of the dual process model suggests that our participants are likely to still be oscillating between the sense-making and meaning-making phases, rather than having fully arrived at high levels of personal growth (which tends to be seen some years after the loss) [24]. It is also likely that a number of variables such as family background, social context, coping skills and individual differences in characteristics, such as resilience and optimism may impact on parents’ course of bereavement. These differences and the concept of oscillating between sense-making and meaning-making have important implications for clinical practice and the provision of support to the bereaved after a suicide.

According to Stroebe and Schut [27,28], women may appear to be more loss-oriented following bereavement, and males more restoration-oriented. With our participants it was not possible to detect obvious differences in responses due to the qualitative nature and small sample size of the study. While several mothers specifically indicated acceptance of their loss (sense-making) at both six and 12 months, both mothers and fathers went on to describe meaning-making, or restoration orientated responses at 12 months.

### Methodological Considerations

In the current study only parents bereaved by suicide were included. Nevertheless, a qualitative study by Harper et al. [29] compared different mothers bereaved by sudden death and found that their experiences were similar, and therefore concluded that they could be considered as a homogenous group. Jordan and McIntosh [30] indicate that some aspects of bereavement are universal across different types of deaths, especially between suicide-bereavement and sudden and violent deaths. While the majority of available studies have used convenience samples of people bereaved over different periods of time, the main strength of our study is the use of a purposive sample to compare grief reactions of parents at two specific time points—six and 12 months after bereavement. However, a study over a longer time period would enable a clearer picture of parental reactions over the long term. Another strength of our study was the ability to compare equal numbers of males and females; as much bereavement research is based on mainly female data due to difficulties in obtaining male participants [31]. It should be noted that although this qualitative approach enabled the collection of rich and detailed data, as with all qualitative research it cannot necessarily be generalized to the wider population.

## 5. Conclusions

Despite the uniqueness of each bereavement story, our study identified three key themes in relation to parental responses to suicide bereavement. The phases of sense-making and meaning-making experienced by participants and the range of both adaptive and maladaptive coping strategies applied are very well explained by the dual process model of bereavement [27], indicating that adapting to bereavement is a dynamic and fluctuating process. The results of this study may indicate hope for parents bereaved by suicide; that their traumatic loss may also present new pathways to personal growth, stronger relationships and a greater appreciation for life.
